# Integrating magnetic capabilities to intracellular chips for cell trapping

**DOI:** 10.1038/s41598-021-98095-5

**Published:** 2021-09-16

**Authors:** María Isabel Arjona, Consuelo González-Manchón, Sara Durán, Marta Duch, Rafael P. del Real, Abhinav Kadambi, Juan Pablo Agusil, Mariano Redondo-Horcajo, Lluïsa Pérez-García, Elvira Gómez, Teresa Suárez, José Antonio Plaza

**Affiliations:** 1 Instituto de Microelectrónica de Barcelona, IMB-CNM (CSIC), Esfera UAB, Campus UAB, 08193 Cerdanyola, Barcelona Spain; 2 Centro de Investigaciones Biológicas Margarita Salas, CIB (CSIC), 28040 Madrid, Spain; 3 Instituto de Ciencia de Materiales de Madrid, ICMM (CSIC), Cantoblanco, 28049 Madrid, Spain; 4 School of Pharmacy, University of Nottingham, University Park, Nottingham, UK; 5 Departament de Farmacologia, Toxicologia i Química Terapèutica, Universitat de Barcelona, 08028 Barcelona, Spain; 6 Institut de Nanociència i Nanotecnologia (IN2UB), Universitat de Barcelona, 08028 Barcelona, Spain; 7 Departament de Ciència de Materials i Química Física, Universitat de Barcelona, 08028 Barcelona, Spain

**Keywords:** Lab-on-a-chip, Actuators

## Abstract

Current microtechnologies have shown plenty of room inside a living cell for silicon chips. Microchips as barcodes, biochemical sensors, mechanical sensors and even electrical devices have been internalized into living cells without interfering their cell viability. However, these technologies lack from the ability to trap and preconcentrate cells in a specific region, which are prerequisites for cell separation, purification and posterior studies with enhanced sensitivity. Magnetic manipulation of microobjects, which allows a non-contacting method, has become an attractive and promising technique at small scales. Here, we show intracellular Ni-based chips with magnetic capabilities to allow cell enrichment. As a proof of concept of the potential to integrate multiple functionalities on a single device of this technique, we combine coding and magnetic manipulation capabilities in a single device. Devices were found to be internalized by HeLa cells without interfering in their viability. We demonstrated the tagging of a subpopulation of cells and their subsequent magnetic trapping with internalized barcodes subjected to a force up to 2.57 pN (for magnet-cells distance of 4.9 mm). The work opens the venue for future intracellular chips that integrate multiple functionalities with the magnetic manipulation of cells.

## Introduction

The progress on miniaturizing semiconductor technologies allows the fabrication of chips at the scale of living cells^1^. These devices emerge as inorganic semiconductor biointerfaces^2^, which can be internalized in a cell, to enable a direct actuation or interrogation of intracellular physical and chemical parameters^3^ from a non-conventional perspective. These chips have dimensions on the range of few microns, augmenting the difficulty, even for microelectronics-based techniques, to integrate functionalities in those small volumes. Despite this difficulty, (bio)chemical sensors coating intracellular silicon chips with vital dyes^4^ or suspended planar array chips for intracellular pH change detection^5^ have been demonstrated. Additionally, intracellular mechanical sensors for mechanobiological studies, such as pressure sensors for osmotic shock characterization^6^ or force sensors for tracking cytoplasmic mechanical properties changes and intraembryonic forces during early fertilization process on mouse embryos^7^, have also been validated. Alike, microcoils, as potential passive radio-frequency identification tags, have been positively internalized by living cells^8,9^. Moreover, there is increasing evidence that phagocytosis appears to be a common feature to many non-professional phagocytic cell lines^10^ expanding the capabilities of these intracellular chips.

Despite their potential, the applications of these devices are at their infancy and not exempt from multiple limitations. For instance, experiments that co-culture cells with chips do not commonly guarantee that all the cells have an internalized chip. For many studies this might not be a limitation, but it can be a true challenge for studies that required the analysis of those exclusive cells that have an internalized device (i.e. devices that induce a biochemical response on the cell). The isolation of cellular subpopulations, cell enrichment, is a common task in cell biology. Among the cell separation techniques, the magnetic selection using magnetic beads for cell enrichment is simple and effective^11^. The magnetic manipulation of cells offers great advantages: (a) magnetic fields penetrate non-magnetic materials (e.g. glass and some polymers) that are commonly used in biological experiments, (b) it is a non-contact technique, and (c), although cells are typically not magnetic, they can be labelled by magnetic beads to exert magnetic forces^12^.

A great advantage is that microchips could benefit from the micro- and nano-electromechanical systems (MEMS and NEMS) technologies to integrate multiple functionalities in a single device. In this sense, the integration with magnetic materials could expand their capabilities into living cell manipulation. We have previously employed polysilicon, a material widely used in microelectronics, to develop miniaturized barcodes that can be used for individual cell^13–15^ and living embryo^15–19^ identification without cell viability interfering. The combination of the functional features of both, microbarcodes and magnetic capabilities, may provide the means to develop devices for the identification of a subpopulation of cells coupled with the capability to manipulate them by an external magnetic field.

Electroplating is one of the methods to deposit and eventually integrate magnetic materials into microfabrication processes. Besides being a cost-effective technique, the whole process requires low energy, and a precise deposit thickness could be accomplished by controlling the deposition charge meanwhile handle complex geometries^20^. The electroplating set up is also easy to maintain and, importantly, the final deposit properties can be adjusted by modifying the solution composition.

We present here Ni intracellular magnetic chips for tagging and trapping subpopulations of HeLa living cells as a demonstration of the capacity of the intracellular chips to integrate cell manipulation capabilities.

## Results

### Design of the magnetic barcodes

**Figure 1:**
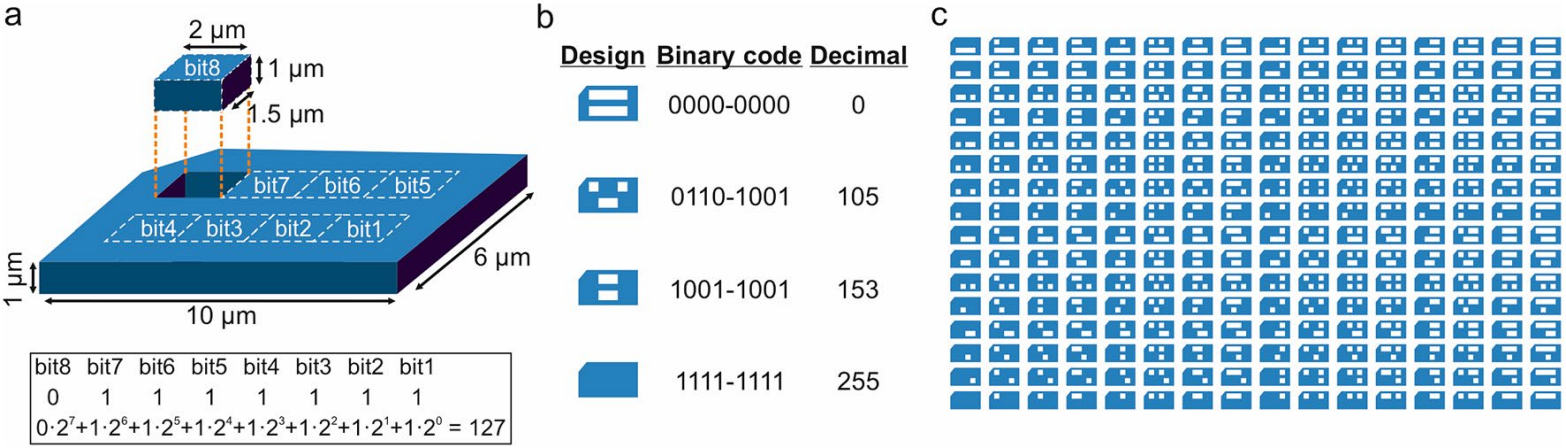
Designed barcodes. (**a**) Schematic of the barcodes comprising 2 rows of 4 rectangles areas representing bits. The presence of material in these areas indicates a ‘1’ and the absence indicates a ‘0’. A mark (cut) on the top left corner indicates the start of the reading position. (**b**) Representation of the physical design, and the corresponding binary and decimal codes for some of the tested devices. (**c**) Designed barcodes showing all possible encoded combinations, from 0 (top right) to 255 (bottom left).

The microbarcode consists of a device with external lateral dimensions fixed to 10.0 µm in length, 6.0 µm in width and 1.0 µm in thickness, in order to make them easily identifiable under an optical microscope. Their encoding labelling is based on a two-dimensional matrix of 2 rows and 4 columns of individual bits (Fig. 1a)^14^, with bit lateral dimensions fixed to 2.0 µm in length and 1.5 µm in width. A solid-bit represents the value 1 (Bit = 1) and a hole-bit represents the value 0 (Bit = 0). A marker in the top left corner is included in the design in order to expedite the correct reading of data. The details of some of the codes used during this study are shown in Fig. 1b (henceforth, a specific barcode will be designated by its decimal representation). Lastly, the complete range of possible designs (2^8^ = 256 codes) can be seen in Fig. 1c.

**Figure 2:**
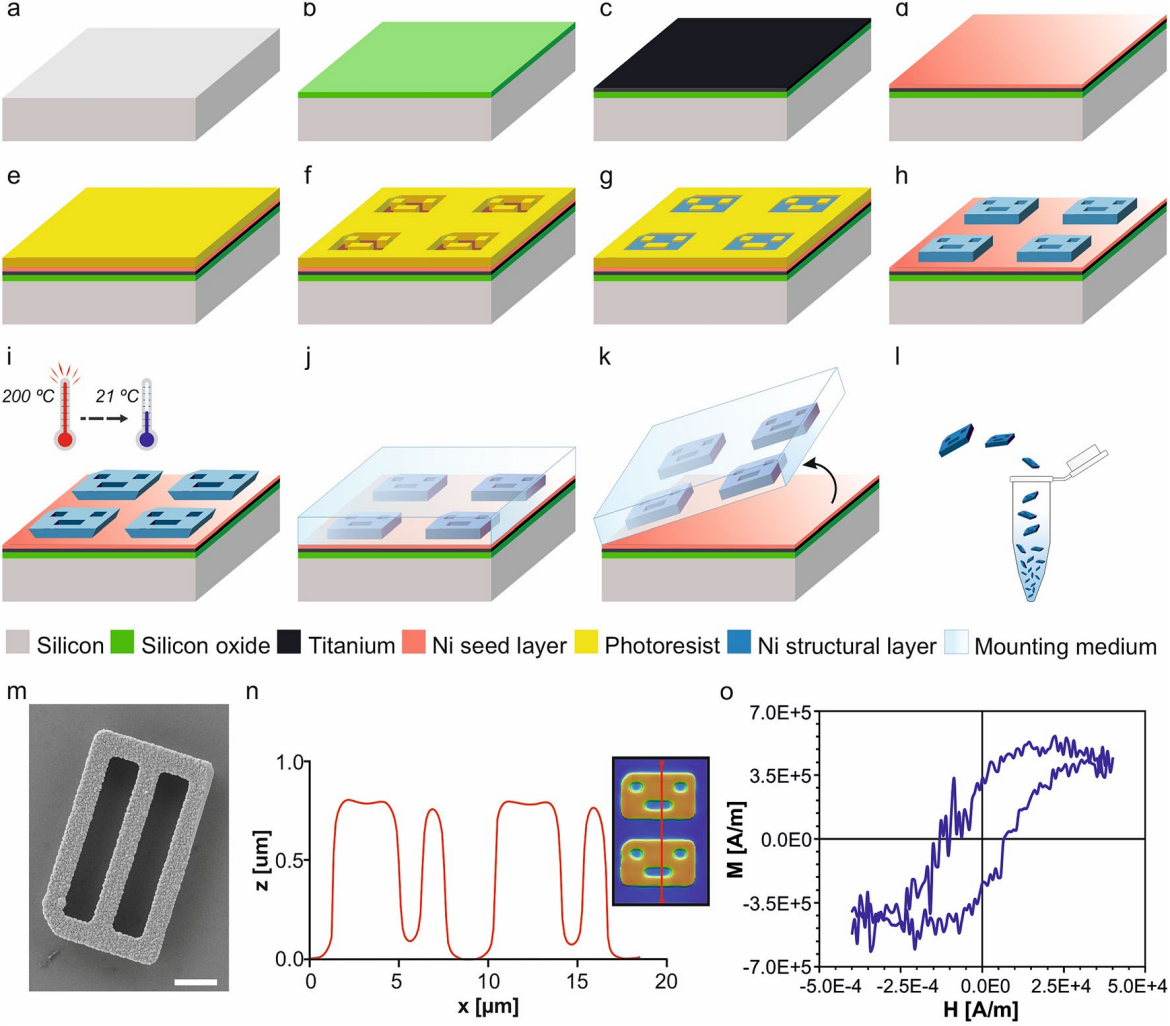
Technology development of the Ni barcodes. (**a**) A silicon wafer substrate as the starting material for the fabrication of the magnetic barcodes. (**b**) 1 µm of silicon oxide was deposited. (**c**) A 50-nm-thick of Ti and (**d**) a 50-nm-thick of Ni were evaporated as metallic seed layers. (**e**) 0.9 µm of photoresist was spun over the surface. (**f**) Patterned photoresist after exposure to ultraviolet light through a photolithographic process to get the inverted barcode pattern. Barcode devices were fabricated from the inverted photoresist pattern. (**g**) 1 µm of Ni was deposited with the electroplating process. (**h**) Removal of the photoresist. (**i**) Thermal-shock was performed after heating the devices at 200 °C for 20 min and followed by a quick cooling down to room temperature. (**j**) A drop of an aqueous mounting medium was cast over the devices. (**k**) Manual peeling of the solidified membrane was performed to release the barcodes. (**l**) Chips were collected in an Eppendorf by dissolving the membrane in water. (**m**) SEM images of a Ni fabricated barcode with code number 0000–0000 = 0, after its release. Scale bar: 2 µm. (**n**) Confocal profiles of Ni barcodes (right inset shows the scanned line), z represents the vertical dimension and x the length along the scanned line. (**o**) Hysteresis loop of Ni barcodes measured with a VSM for magnetic characterization. *M* represents the magnetization and *H* is the applied magnetic field.

Our previous polysilicon barcodes used for cell labelling were fabricated using a photomask with positive patterns of the codes to transfer the design via photolithography directly onto the substrate^13,14,16–19^. In the present work, it was necessary to first obtain an inverted barcode photoresist mould (Fig. 2a–f), as the electrodeposited magnetic layer will be subsequently grown to fill the voids of this mould (Fig. 2g).

### Development of an electroplating process to integrate magnetic capabilities

We explore a Ni-based electroplating process to build our devices as this technique has often been used for MEMS fabrication. The fabrication of the magnetic barcodes started with a 100-mm p-type silicon wafer as substrate (Fig. 2a), where a 1-µm-thick silicon oxide (SiO_2_) layer was later deposited (Fig. 2b). An initial 50-nm-thick Titanium (Ti) layer and a second 50-nm-thick Ni layer were evaporated as seed layers to allow the further deposition of the Ni layer (Fig. 2c,d, respectively). Then, a 0.9-µm-thick photoresist coat (Fujifilm OiR 620-09) was spun (Fig. 2e) and finally patterned by photolithography to obtain a well-defined engraved pattern of the devices on the wafer (Fig. 2f). Thicker photoresist layers would result in the potential diminishing of device features.

Ni layer deposition (Fig. 2g) was conducted starting from substrates with the engraved photoresist pattern (Fig. 2f). Afterwards, a Watts nickel electroplating bath was used to optimize the deposit of Ni onto the exposed areas of the substrates. The Watts Ni plating process consisted of an aqueous bath of 280 g/L of nickel(II) sulfate hexahydrate (NiSO_4_·6H_2_O) and 45 g/L of Nickel(II) chloride hexahydrate (NiCl_2_·6H_2_O), and 30 g/L of boric acid (H_3_BO_3_) to maintain the pH around 4 and as a reducer in the hydrogen reaction (“Materials and methods”).

For the set-up, a system of two electrodes (“Materials and methods”) was adequate to achieve an accurate Ni deposit, under an optimized working current density of 30 mA/cm^2^, for a fixed current of 15 mA during 40 s at room temperature (RT). Afterwards, the photoresist was stripped (Fig. 2h).

The fabricated magnetic barcodes had to be released from the substrate in order to get them in suspension for their applicability. Previously, we have released microfabricated polysilicon barcodes by a wet etching process of the sacrificial SiO_2_ layer with hydrogen fluoride vapour (HF 49%)^14^. Here, there were three layers below the Ni devices: SiO_2_, Ti and Ni layers. The SiO_2_ could be used as sacrificial layer, however the HF vapours damaged the Ni devices. Hence, the challenge of releasing the chips from the wafer was optimized by following a two-step process. First, a thermal shock was done by heating to 200 °C and then a rapid cooling down to 21 °C (Fig. 2i) to weaken the adhesion at the interface, namely between chips and the nickel seed layer. Afterwards, a highly effective peel-off method^5,21^ was used (Fig. 2j,k, “Materials and methods”). The peeled membrane with the embedded fabricated devices (Fig. 2k) was dissolved in deionized (DI) water and chips were collected in Eppendorf tubes by centrifugation (Fig. 2l). In order to avoid the contamination of the chips, which can ruin the posterior biological test, the DI water was replaced by 96% ethanol during storage, collecting about 0.43 million of Ni barcodes per Eppendorf.

### Barcodes characterization

After the release step, Ni barcodes were initially characterized by SEM inspection, showing a well-defined shape and accurate dimensions (Ni barcode 255, Fig. 2m). Additionally, the device thickness was evaluated by confocal microscopy (“Materials and methods”) before their release from the wafer, showing a thickness of 0.81 µm (Fig. 2n, Ni barcode 105).

The magnetic characterization of the barcodes was performed by the use of a vibrating sample magnetometer (VSM). The test shows the hysteresis loop of the magnetization, *M*, of an array of Ni barcodes, Fig. 2o. Devices were spread on a piece of silicon wafer and a saturating magnetic field of 1.8 T was applied to know the maximum magnetic moment of the array. Although the measurement resulted noisy due to the small size of the samples (amount of Ni), we managed to obtain the *M* curve close to the detection limit of the equipment.

The magnetic moment of a single barcode, ${m}_{Ni}$, that posteriorly will be used to know the force under a magnetic field gradient, can be calculated following the Eq. (1) for Ni barcodes^22^:

1 $${m}_{Ni}={M}_{Ni}\cdot V,$$

where ${M}_{Ni}$(${{\mu }_{0}M}_{Ni}=0.61 T$) is the saturation magnetization of Ni and $V$ is the volume of the barcode, being *µ*_*0*_ the vacuum magnetic permeability. The magnetic moment of the barcodes depends on their volume [see Eq. (1)]. The maximum volume corresponds to the barcode 1111–1111 = 256 (*V*_*max*_ = 60 µm^3^) and the minimum to the barcode 0000–0000 = 0 (*V*_*min*_ = 36 µm^3^). A mean value of the volume corresponds to all the barcodes which has 4 bits with value ‘1’, such as the 0110–1001 = 105 or 1001–1001 = 153 (*V*_*mean*_ = 48 µm^3^). These volumes correspond to 29.1 × 10^–12^, 17.5 × 10^–12^ and 23.3 × 10^–12^ A m^2^ magnetic moments, respectively.

### Biological validation of Ni barcodes

**Figure 3:**
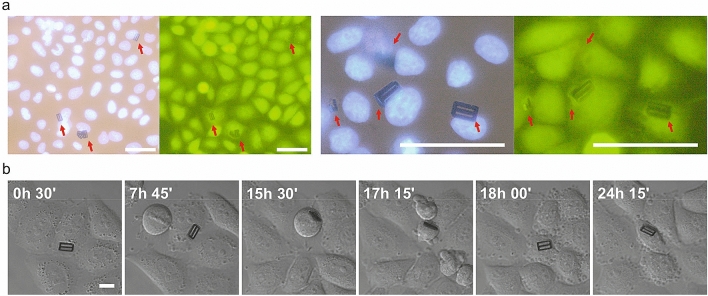
HeLa cells viability and tagging with Ni barcodes. (**a**) Images of two samples of fixed HeLa cells stained with DAPI (nuclei, blue) and the vital dye CellTracker (green fluorescence), with different magnification. Red arrows point the position of the Ni barcodes. Scale bars: 40 µm. (**b**) Images taken from a 24-h videomicroscopy showing a HeLa cell tagged with a Ni barcode inside (code number: 0000–0000 = 0). Cell before division (15 h 30′) and daughter cells (17 h 15′). Scale bar: 10 µm.

The intended uses of the suspended Ni barcodes encompass living cell tagging and cell manipulation. Thus, HeLa cells were grown and co-cultured with the barcodes following a protocol to facilitate cell-device contact as well as microscopic analysis (“Materials and methods”). Despite HeLa cells are non-professional phagocytes, we observed by confocal microscopy the internalization of the magnetic barcodes, in agreement with the reported fact that non-professional phagocytosis could be a general feature to normal tissue lines^10^. The viability of Hela cells with internalized Ni barcodes was assessed using a fluorescent vital dye labelling (“Materials and methods”), where barcode-bearing HeLa cells appeared viable and identical to neighbouring cells (Fig. 3a).

The capabilities of the internalized barcodes to label a subpopulation of these cells was also demonstrated by monitoring them under an inverted microscope together with a CCD camera (Fig. 3b). For instance, a labelled cell with a Ni barcode was identified (barcode number: 0000–0000 = 0) and tracked for more than 24 h (Fig. 3b), showing that the cell underwent division cycles comparable to those of the surrounding control cells. After division the chip remained with one daughter cell.

### Non-contact cell trapping by external permanent magnet

After demonstrating their biological compatibility and tagging capability, the Ni barcodes were tested for non-contact magnetic trapping of a subpopulation of HeLa cells. Gradients of magnetic fields are typically applied for microobjects trapping. The microobjects are moved towards the magnetic source and retained in a specific place where the maximum value of the magnetic field is located^12^. Magnetic trapping is also commonly used in cell biology by tagging the cells with magnetic beads. Thus, we aim to incorporate this capability to the intracellular chip technologies.

**Figure 4:**
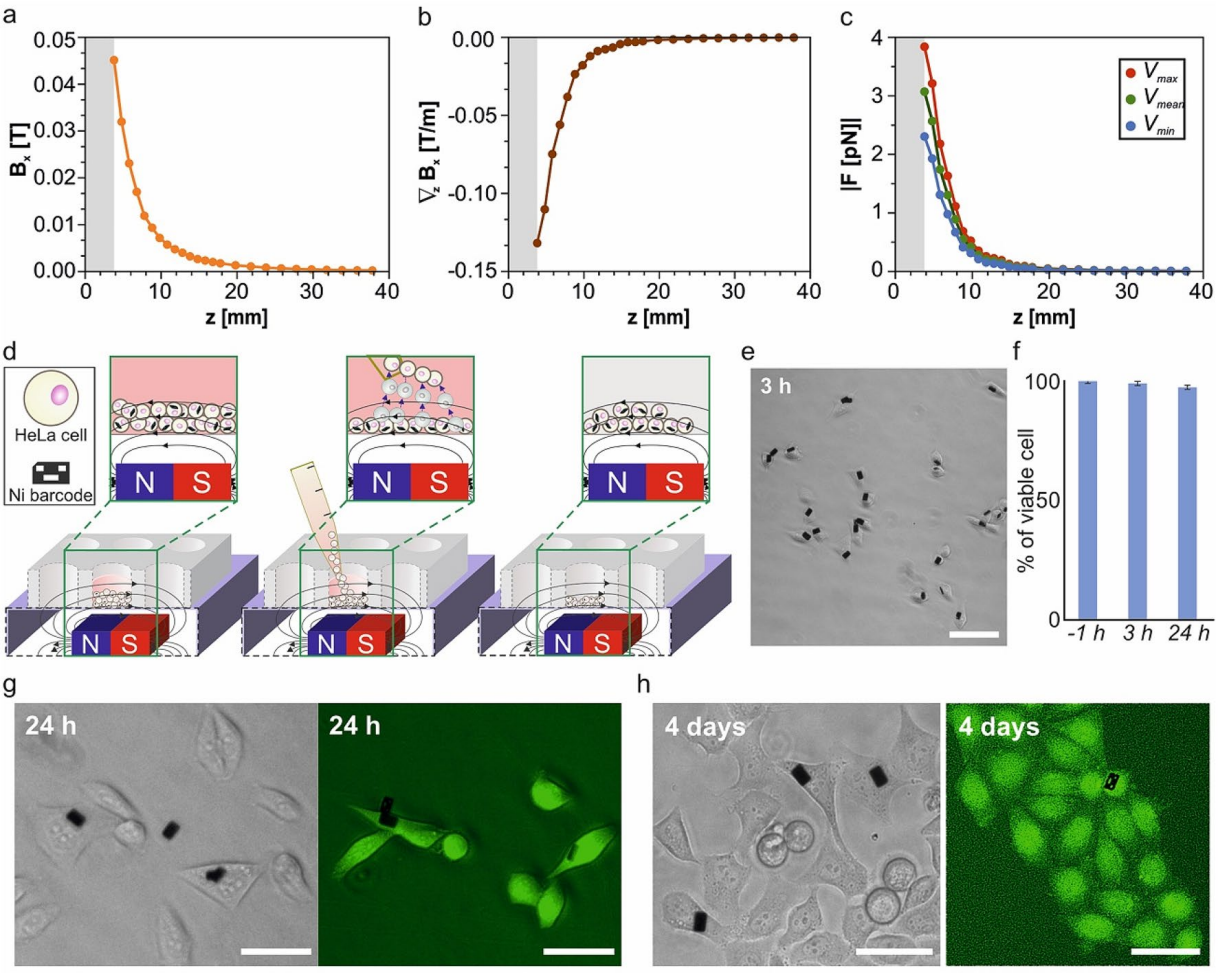
Magnetic trapping of the subpopulation of HeLa cells with internalized Ni barcodes. (**a**) Measured magnetic field, ${B}_{x}$, of the magnet and (**b**) the calculated gradient of magnetic field, ${\nabla }_{z}{B}_{x}$, versus the distance to the magnet, *z*. (**c**) Estimated force, *F*, applied over the Ni devices (assuming magnetic saturation) depending on the distance to the magnet for the maximum, mean and minimum barcode volumes. Measurements for Hall probe-magnet distances below 3.81 mm (grey area) were not possible to carried out due to the position of the Hall sensor inside the probe. (**d**) Schematic representation of the cell separation process. (left) A magnet was placed close to the bottom (4.9 mm) of the well containing the cell suspension. After a 15 s exposure, cells with internalized magnetic barcodes are retained and trapped, named positive selection, and cells without a device could be transferred to another well (center), thus enriching the cell population with internalized Ni barcode that remains in the first well. (**e**) Optical image of HeLa cells 3 h after magnetic separation. (**f**) Viability of cells cultured with Ni barcode before (− 1 h) and after (3 h and 24 h) magnetic separation. Percentage values were obtained by analyzing between 300 and 1000 cells for each condition, mean and SD, *n* = 4. (**g**) and (**h**) Optical and EFM images of HeLa cells stained with 2 µM calcein-AM (green fluorescence) 24 h and 4 days after magnetic separation, respectively, to assess cell viability. Scale bars: 40 µm.

To reduce the impact on the typical set-ups used by cell biologists, we propose to use permanent magnets in our system, which produce high magnetic fields meanwhile avoid the use of an external energy source. We constructed a magnetic set-up placing a magnet under the well dish containing the cell culture. The magnetic field of the magnet, $\overrightarrow{B }={\overrightarrow{{u}_{x}}B}_{x}$, was determined, (Fig. 4a, “Materials and methods”) in order to determine the gradient of this field, ${\overrightarrow{\nabla }}_{z}{B}_{x}$ (Fig. 4b). The magnetic force, ${F}_{z}$, acting on a single barcode (and therefore on the cell) can be expressed by:

2 $${F}_{z}={m}_{Ni}\cdot {\nabla }_{z}{B}_{x}.$$

The magnetic force on the barcode was calculated depending on the design (binary code) (Fig. 1) and it depends on its volume. The magnetic force will be exerted on the cells by the internalized chips. This force could reach 3.83 pN at a distance of 3.81 mm for barcode 255 with the maximum magnetic moment (Fig. 4c), assuming that the barcode is magnetically saturated. The magnetic force curve shows the characteristic decreases with the distance to the magnet (Fig. 4c).

To demonstrate the cell manipulation capabilities, HeLa cells were initially seeded and cultured with Ni barcodes for 24 h, with different cell-chip ratios. We selected the 2:1 ratio, where between a 5–13% of cells were observed with internalized barcodes, a percentage where any cell enrichment of cells with internalized chips will be easily detected. Also, with the 2:1 ratio, the number of cells with more than one chip was negligible.

To proceed with the cell enrichment, it was required a previous process of cell dissociation from the substrate of culture, a process called trypsinization. HeLa cells are adherent cells whose adhesion forces to the substrate can be at the micronewton range^23^. These adhesion forces are 3 orders of magnitude larger than the maximum magnetic forces that we can generate by the Ni devices. In addition, the lack of adhesion is also a conventional process in cell biology required to remove non-trapped cells by pipetting. Thus, in our experiments, cells were detached with 0.05% trypsin for 1 min.

The previously characterized magnet was located 4.9 mm under the well dish to perform the magnetic separation (Fig. 4d, Materials and methods). Therefore, among the cell-barcode pairs, those that are magnetically saturated will endure magnetic forces of ~ 2.57 pN, while for those that are not saturated this value will be smaller (Fig. 4c). Larger forces could be obtained by locating the magnet closer to the well dish containing the cell culture. However, due to the hysteretic behaviour of the barcodes, they present a non-zero magnetization, even at zero applied field. Therefore, they behave like magnetic gradient generators that (if they are close enough) could produce significant forces between them favouring the aggregation of the cells. Hence, we avoided that situation which could interfere with the subsequent cell manipulation.

Detached HeLa cells, with and without internalized Ni barcodes, were exposed for 15 s to the magnetic field (Fig. 4d). This process allows the trapping of the HeLa cells with magnetic devices at the bottom of the well, meanwhile cells without magnetic devices were removed gently by micropipette aspiration. Then, fresh medium was added to the trapped cells and the whole process was repeated to allow two enrichment cycles. Three hours after magnetic separation, these cells looked healthy and firmly attached to the plate (Fig. 4e) confirming an enrichment in the population of barcode-bearing cells from 6 to 60%.

Significantly, cell viability experiments after 3 h and 24 h of culture showed similar results to that of the cells before separation (Fig. 4f,g). Moreover, 4 days after magnetic separation HeLa cells which bear Ni barcodes appeared healthy (Fig. 4h), and, as expected, diluted due to the expected cell culture proliferation.

## Discussion

In this work, we have demonstrated the magnetic manipulation of living cells by intracellular chips. After trypsinization, HeLa cells with internalized chips were trapped by the magnetic field gradient generated by an external magnet and a cell enrichment from 6 to 60% in 2 cycles was demonstrated. Magnetic targeting is an emerging method in experimental medicine that is being used, for instance, to dock manipulated immune cells loaded with magnetic beads^24^ to a specific target, such as a tumor, through the application of an external magnetic field^25^. A major drawback of magnetic nanoparticles, mostly super-paramagnetic iron oxide nanoparticles, used in these procedures, is the increased toxicity of the cellular charge of nanoparticles necessary to infer enough magnetism to the cell^26–29^. Ni-based intracellular chips at the micron scale showed no effects on the viability of HeLa cells, they correctly divide, and one single Ni microchip exerts enough magnetic force to manipulate a non-adhered HeLa cell. In contrast, 4 million of Fe_3_O_4_ (Ms = 0.48 106 A/m) nanoparticles with a diameter of 30 nm (representative diameter of nanoparticles) would be needed to generate the same magnetic forces within a single cell.

Compared with chemically synthesized micro- and nanoparticle technologies, intracellular chips share the advantages of the MEMS and NEMS technologies. A relevant benefit is the possibility to integrate multiple functionalities in the same chip. Based on photolithographic processes, we patterned the chips to obtained magnetic microbarcodes and demonstrated tagging and magnetic trapping functions in a single chip. Our cellular barcoding strategy, based on microscope-readable nickel magnetic barcodes, is a proficient candidate to tag and manipulate living cells.

Although cell-specific and more comprehensive toxicity and biological tests will be required, particularly to properly assess the long-term impact of the chips on cellular function, the possibility to magnetically manipulate cells offered by intracellular chips could open new prospects in many technologies related to fundamental cell biology and health. For instance, many areas of basic research in these fields use cell separation or sorting technologies^25,30–32^. The fact that the presented technology offers the possibility to produce intracellular chips with different volumes, which could cause magnetic forces of different values, could open the venue to use magnetic chips for cell separation and sorting. To explore the possibility of performing cell separation, individual cell shape and size will be relevant parameters in extensive fluid dynamic studies. It is important to notice that, the proposed trapping technology, as any other one based on magnetic fields, should be evaluated for those cells showing intrinsic magnetic behaviour, since barcodes could interfere in the magnetic properties of the cells, and the cells themselves would contribute to the force through their magnetic moment. Thus, our results could be a first step in the development of new technologies based on the separation and/or the magnetic vectorization of tagged living cells. Another example is single cell analysis, where there is a promising set of technologies focused to determine the heterogeneity of a population of cells^33^. Intracellular magnetic chips, accompanied with the development of substrates with localized magnetic fields, could contribute to individual cell trapping to favour the analysis of an individual cell.

Overall, the major advantage of the intracellular chips is the integration capability of their fabrication technologies. Mechanical, biochemical, chemical, electrical and electromagnetical chips have been already internalized and validated without affect the cell viability^4–9,34^. Thus, we envision the integration of magnetic manipulation capabilities into chips that could integrate multiple functionalities as a key element in the field of intracellular chips.

## Materials and methods

### Materials

Nickel(II) sulfate hexahydrate (NiSO_4_·6H_2_O, Merck) and Nickel(II) chloride hexahydrate (NiCl_2_·6H_2_O, Merck) and boric acid (H_3_BO_3_, Panreac) were used in the Watts bath for Ni platting process. Aqueous mounting medium (Fluoromount, Sigma-Aldrich) was used for device releasing. CellTracker (Molecular Probes) and calcein-AM (Thermofisher) were used as vital stains. Cell nuclei was stained with DAPI (Merk). Fluoromount-G (SouthernBiotech) was used for cell mounting to microscopy analysis. A VSM (KLA Tencor EV7, LOT-Oriel) was used to obtain the hysteresis loop of the devices at the ICMM (CSIC). SEM (Auriga Microscope, Carl Zeiss GmbH) was used for device analysis and characterization. Confocal microscopy (PLu neox, 3D optical profilometer, Sensofar) provides topographic profiles of the barcodes.

### Watts bath for Ni layer deposition

The aqueous bath for the Ni platting process consisted of 280 g/L of nickel(II) sulfate hexahydrate (NiSO_4_·6H_2_O) and 45 g/L of Nickel(II) chloride hexahydrate (NiCl_2_·6H_2_O). 30 g/L of boric acid (H_3_BO_3)_ were added to keep the pH around 4, also acting as a reducer in the hydrogen reaction. Nickel sulphate was the main Ni supplier to the sample, given that it is easily soluble (as chloride) and a non-complex ion source. The nickel chloride improved the quality of the deposit since it allowed a high current density limit due to an increased ion diffusion coefficient and helps to avoid the passivation of the anode. The two electrodes set-up comprise an anode of nickel and a cathode which consists in a 0.5 cm × 1.0 cm piece of wafer to deposit the Ni layer (Fig. 2g). Both electrodes were connected to the potentiostat (Autolab 302 N, Metrohm, AG) at a fixed working current of 15 mA during 40 s at RT, that results a current density of 30 mA/cm^2^. For the experiments we have performed sample batteries in freshly prepared baths. Within each battery, the parameters of the composition (temperature and current intensity) are conserved, thus the fabricated samples resulted in reproducible device dimensions.

### Releasing of fabricated barcodes

The releasing process was a two-step process. Initially, a thermal shock was done by placing the samples in an oven for 20 min at 200 °C and their subsequent rapid cooling to 21 °C (Fig. 2i). The exposure to the high temperature induced an initial thermal expansion while the rapid cooling forced the contraction of the layers on the wafer. This expansion and contraction increased the material stresses, weakening the adhesion at the interface, namely between chips and the nickel seed layer. A drop of an aqueous mounting medium was first placed directly on top of the anchored chips and left to solidify at room temperature (RT) (Fig. 2j). A subsequent manual force was used to peel the solidified, flexible membrane encircling the chips (Fig. 2k). Finally, chips were collected in Eppendorf tubes by centrifugation after dissolving the water-soluble membrane (Fig. 2l). The centrifugation of the released devices was performed at 5000 rpm for 5 min to avoid damaging the barcodes. Three changes of medium to assure that the polymer was completely removed, where the last of these was replaced by 96% ethanol for a sterile storage of the devices.

### Cell culture for viability assay

HeLa cells were cultured with DMEM medium (Gibco) in standard conditions, at 37 °C and 5%CO_2_. For viability assays, Hela cells were grown on glass coverslips at a confluence of 40–50% and, after 24 h, different Ni barcodes amounts ranging from 2500 to 10,000 were added in different experiments, and incubation continued another 24 h. To analyse cell viability, cells were stained with the vital dyes CellTracker (1 µM) or Calcein (2 µM) for 45 min used as indicated by the manufacturer) and, then, fixed with 4% PFA and the nuclei stained with DAPI. Cells were mounted with Fluoromount-G and analysed under the microscope.

### Set-up for the magnetic cell separation

To demonstrate the cell manipulation capabilities, 50,000 HeLa cells were initially seeded in triplicate in a 12-well plate and cultured with 25,000 Ni barcodes (ratio cell-chip 2:1) over 24 h. Then, cells were detached with 100 µL of 0.05% trypsin and, after adding 0.5 mL of complete medium, were exposed to the magnetic field. The magnet was located beneath the plate to hold cell-bearing Ni barcodes on the bottom while removing the rest of the culture. A gaussmeter Lakeshore 475 DSP was used to measure the magnetic field along a perpendicular plane to the magnet, $(\overrightarrow{B }={\overrightarrow{{u}_{x}}B}_{x})$. For the calculations to find the maximum forces acting on the barcodes, we always assumed magnetically saturated chips. These forces will diminish when the magnet-device distance increases (they are no longer magnetically saturated), but due to the fact that experimental magnetic forces were enough to manage the cell trapping, it was not necessary to reach saturation.

### Image collection and analysis

Optical and fluorescent images were taken under a DMI6000B inverted Leica microscope coupled to a Hamamatsu CCD 9100-O2 camera with time-lapse technology and using a ZEISS Axioskop microscope coupled to a Leica DFC 400 camera and a Leica DM IL3 microscope coupled to a Leica DFC 3000 G. Objectives 40× and 63× were regularly used and barcodes clearly read with the 63× objective. Confocal microscopy to assess that chips were inside the cells was performed with a TCS SP5 AOBS CLSM (Leica Microsystems GmbH).
